# Case Report of Disseminated Adrenal Histoplasmosis and Secondary Adrenal Insufficiency

**DOI:** 10.7759/cureus.30614

**Published:** 2022-10-23

**Authors:** Ishitha Jagadish, William J Chen, Reema Agarwal, Ramy Shoela

**Affiliations:** 1 Department of Diagnostic Radiology, Saint Louis University School of Medicine, Saint Louis, USA

**Keywords:** immunocompromised, secondary adrenal insufficiency, disseminated histoplasmosis, histoplasma capsulatum, adrenal histoplasmosis

## Abstract

Histoplasmosis is a chronic, infectious disease caused by the environmental fungus *H. capsulatum*, primarily affecting the respiratory system. In immunocompromised patients, histoplasmosis can become severely complicated due to dissemination into various other organ systems. Adrenal insufficiency is an uncommon complication of disseminated histoplasmosis, as its manifestation requires necrotizing granulomatous inflammation of both adrenal glands. We describe a rare case of delayed histoplasmosis in the bilateral adrenal glands and liver of an immunocompromised patient with development of symptoms at 21 years after liver transplant and nine years after renal transplant. In addition, this patient presented with secondary adrenal insufficiency due to long-term use of corticosteroids rather than the typical primary adrenal insufficiency seen in histoplasmosis with adrenal involvement.

## Introduction

Histoplasmosis is a chronic, infectious disease that primarily affects the respiratory system and is the most common fungal infection in the United States. It is caused by the *Histoplasma capsulatum species*, known to be endemic to the Ohio and Mississippi river valleys. However, *H. capsulatum* may survive in other geographic areas due to factors such as human migration patterns and climate change. From 1938 to 2013, over 100 outbreaks of histoplasmosis involving around 3000 cases of incidence were reported in 26 states and the territory of Puerto Rico. *H. capsulatum* is a dimorphic fungus that exists as mold in the environment and yeast-like structures with septate hyphae at 37 degrees Celsius in tissues. Exposure to *H. capsulatum* can occur if persons come into contact with soil enriched in the excrement of birds and bats through spelunking in caves or working on construction sites [1].

The clinical presentation of disseminated histoplasmosis is dependent upon the immune function of patients and their degree of exposure to the fungus. Most infections with *H. capsulatum* occur in immunocompetent persons as an asymptomatic and self-limited respiratory infection. Substantial exposure to *H. capsulatum* may result in symptomatic pulmonary histoplasmosis even in immunocompetent persons. Acute infection tends to occur in pediatric and/or immunocompromised patients, with the clinical presentation involving fever, fatigue, pancytopenia, and hepatosplenomegaly. Less common signs and symptoms include diarrhea and dyspnea. The course of infection in these more vulnerable patients may be complicated by severe, disseminated histoplasmosis affecting multiple organ systems such as the gastrointestinal, central nervous system, bone marrow, and adrenal glands. Chronic infection tends to present in older, immunocompetent patients as pancytopenia, hepatosplenomegaly, oropharyngeal and/or skin lesions, gastrointestinal involvement, and signs and symptoms of adrenal gland dysfunction [2].

*H. capsulatum* has the capability to remain dormant for years and reactivate later when cell-mediated immunity is diminished by immunosuppressive agents or other disease states. Hence, histoplasmosis should be considered as a differential diagnosis in patients who have previously traveled to or lived in endemic areas [1]. Patients may also present with signs and symptoms shortly after exposure to the fungus or may experience cycles of symptomatic relapses followed by asymptomatic periods [2].

Latent histoplasmosis infections tend to result in poor outcomes for patients with prior solid organ transplantation. A retrospective analysis of 152 histoplasmosis cases in solid organ transplant recipients found that the histoplasmosis-related mortality rate was 10%. Of these deaths, 72% occurred within the first month after diagnosis of histoplasmosis. One-third of the histoplasmosis cases occurred within a one-year post-transplant period, and less than half of the cases occurred in the first two years after the transplant. The longest interval from transplant to histoplasmosis diagnosis was 20 years [3]. The latent period of *H. capsulatum* may be prolonged for up to 60 years in rare cases, as per a case report of an ex-serviceman with chronic disseminated histoplasmosis [4]. Still, the presentation of disseminated histoplasmosis may be similar to other chronic infections or malignancies. A broad list of other differential diagnoses should be considered, including sarcoidosis, tuberculosis, adrenal hemorrhage, metastatic carcinoma, and lymphoma [5].

Adrenal insufficiency is an uncommon complication of disseminated histoplasmosis which may be challenging to manage. Both adrenal glands must have necrotizing granulomatous inflammation for primary adrenal insufficiency to manifest [1]. Patients with adrenal histoplasmosis can present with symptoms of fatigue, weight loss, nausea, and orthostasis for months. Labs, vitals, and physical exam findings of primary adrenal insufficiency may include hypoglycemia, dehydration, orthostatic hypotension, and hyperpigmentation. In the event of adrenal hypofunction, adrenal histoplasmosis should be considered as a differential diagnosis, especially when the adrenal glands are enlarged. In some patients, the adrenal glands have been described as the sole site of actively disseminated histoplasmosis [6].

We present a rare case of delayed presentation with disseminated histoplasmosis in both adrenal glands and liver of an immunocompromised patient. The patient first exhibited signs and symptoms of histoplasmosis around 21 years after liver transplant and nine years after renal transplant. It is unusual to see a significantly prolonged latent period of disseminated histoplasmosis in patients with history of solid organ transplants, and the rarity of such presentations may cause difficulty in diagnosing the etiology. This case demonstrates the importance of considering the activation of dormant histoplasmosis as a part of the differential diagnosis. In addition, the expected presentation of adrenal histoplasmosis is primary adrenal insufficiency; however, our patient was found to have secondary adrenal insufficiency due to prolonged use of corticosteroids.

## Case presentation

A 63-year-old male patient with a past medical history of cirrhosis secondary to Hepatitis C (HCV), orthotopic liver transplant, renal transplant for end-stage renal disease on hemodialysis, and splenectomy presented to a university hospital in the Midwest with a fever of 101.7 ℉ which resolved without medications, chills, fatigue, and weakness. All workup described is also organized into a chronological, tabulated format, including the tests ordered and the results obtained (Table 1). 

**Table 1:** Tabulated format of investigations performed for the case at hand

Test Ordered	Results
Outpatient Magnetic Resonance Imaging (MRI) with MR Cholangiopancreatograph (MRCP)	New peripheral intrahepatic bile duct dilatation; new, hypointense masses in bilateral adrenal glands with peripheral enhancement, suggestion of some septations in the left adrenal gland, no soft tissue stranding, and no significant drop out of phase
CBC	Within normal limits
CMP	Sodium 131 mEQ/L, Bicarbonate 20 mEQ/L, ALT 71 U/L, AST 98 U/L, Total Bilirubin 6.2 mg/dL and continued to rise throughout admission, Gamma-Glutamyl Transpeptidase 116 U/L
Respiratory viral panel	Negative
Urine culture	Negative; no growth
Sputum culture and gram stain	Negative; moderate normal oropharyngeal flora, no fungal organisms
Chest x-ray	No acute abnormality
Computed Tomography (CT) of the chest	Ground glass opacities in the lungs, paraseptal emphysema, and calcified granulomas
*Blastomyces dermatitidis antigen* quantitative test for urine and blood cultures	Negative; not detected
Histological evaluation of CT-guided left adrenal gland biopsy	Intracytoplasmic *Histoplasma* species with background necrosis and acute inflammation
Histological evaluation of ultrasound-guided liver biopsy	Bridging fibrosis in the graft liver is likely related to previous recurrent Hepatitis C infection, granulomatous hepatitis due to *Histoplasma* species, and no evidence of acute cell-mediated rejection
Adrenocorticotrophic (ACTH) stimulation test	Secondary adrenal insufficiency
Repeated CT of the chest	Multifocal pneumonia in bilateral lungs
Esophagogastroduodenoscopy	Esophageal varices with no active bleeding and multiple aphthous ulcers in the esophagus
Urine culture, Sputum culture and gram stain	Positive for *Klebsiella pneumoniae*
Blood culture	Negative; no growth
Cerebrospinal fluid (CSF) cultures	Negative; no growth
Stool test for *Clostridium difficile *antigen and Toxin A + B	Positive for *Clostridium difficile *antigen and Toxin A + B

**Figure 1:**
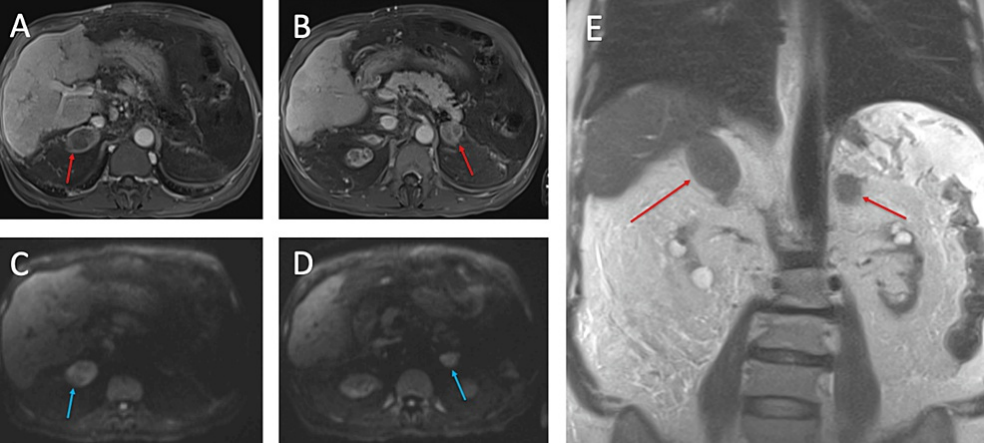
MRI of the abdomen with and without intravenous contrast showing bilateral necrotic adrenal glands Bilateral rim enhancing, centrally necrotic adrenal gland masses (red arrows), measuring (A) 4 cm on the right and (B) 2.9 cm on axial MRI with possible septations on the left. (C, D) The bilateral adrenal gland masses demonstrate diffusion restriction on axial MRI (blue arrows). (E) T2 haste sequence demonstrating T2 hypointense masses in bilateral adrenal glands on coronal MRI.

Before the patient’s presentation, an outpatient Magnetic Resonance Imaging (MRI) with MR Cholangiopancreatography (MRCP) showed new peripheral intrahepatic bile duct dilatation, concerning for secondary sclerosing cholangitis and hepatic transplant failure. New, bilateral adrenal masses with the suggestion of some septations in the left adrenal gland were also noted (Figure 1). These adrenal lesions showed no significant drop out of phase, and the postcontrast study demonstrated peripheral enhancement. There was no soft tissue stranding in the adjacent adipose tissue. A coronal T2 haste sequence of the MRI demonstrated hypointense masses in the bilateral adrenal glands (Figure 1E).

**Figure 2:**
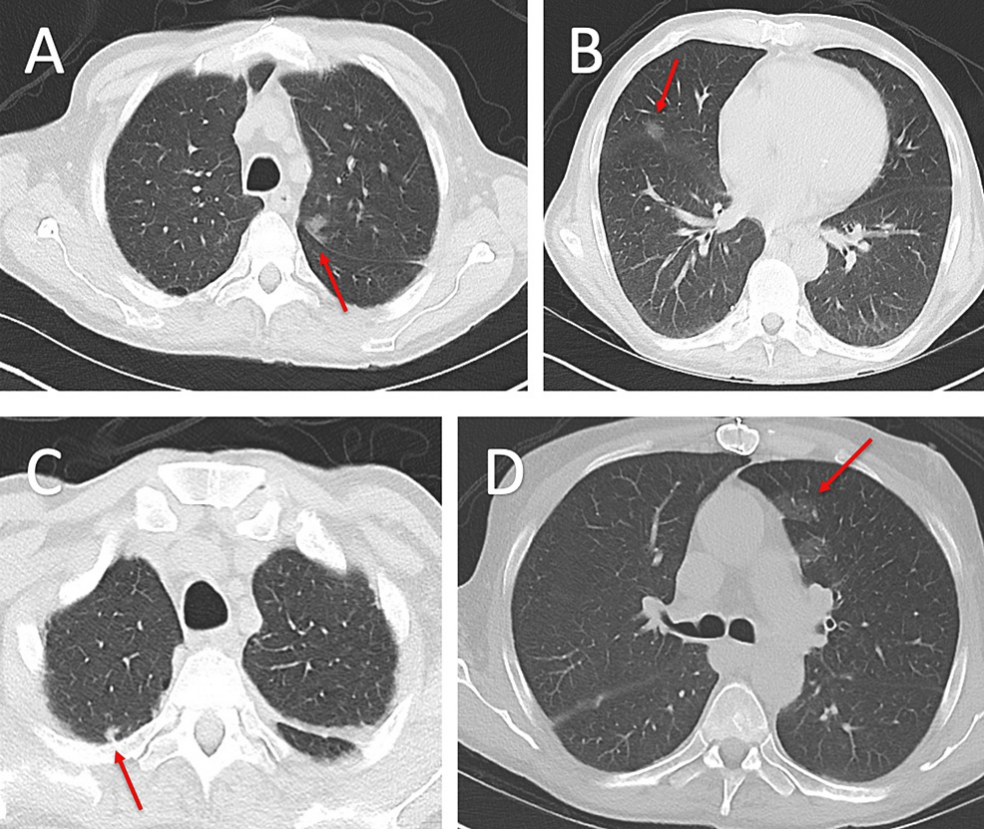
Axial chest CT demonstrates multiple ground glass opacities in bilateral lungs Multiple new-onset ground glass opacities (red arrows) are seen bilaterally in (A) the left upper lobe, (B) along the right horizontal fissure in the right middle lobe, (C) in the posterior right upper lobe associated with the pleura, and (D) in the lingula.

The patient was admitted for the management of sepsis and cholestatic liver injury and endorsed daily adherence to the prescribed immunosuppressive regimen of mycophenolate mofetil, tacrolimus, and prednisone. In the Emergency Department, the patient was afebrile with a heart rate of 84 bpm and blood pressure of 90/59 mmHg. Admission labs were significant for hyponatremia to 131 mEq/L, low bicarbonate of 20 mEq/L, elevated ALT of 71 U/L, elevated AST of 98 U/L, elevated total bilirubin of 6.2 mg/dL, and elevated gamma-glutamyl transpeptidase of 116 U/L. Bedside ultrasonography showed an 8 cm pocket of left-sided abdominal fluid, and a consequent bedside paracentesis was performed. Culture and gram stain of the peritoneal fluid demonstrated the absence of organisms and the presence of rare polymorphonuclear leukocytes, suggesting lower concern for spontaneous bacterial peritonitis. A respiratory viral panel and cultures of the urine, sputum, and blood were all negative. Chest X-ray showed no acute abnormality. Computed Tomography (CT) of the chest demonstrated ground glass opacities in the lungs, paraseptal emphysema, and calcified granulomas which incited concern for multifocal infection (Figure 2). A quantitative *Blastomyces dermatitidis* antigen test conducted on blood and urine samples was negative.

**Figure 3:**
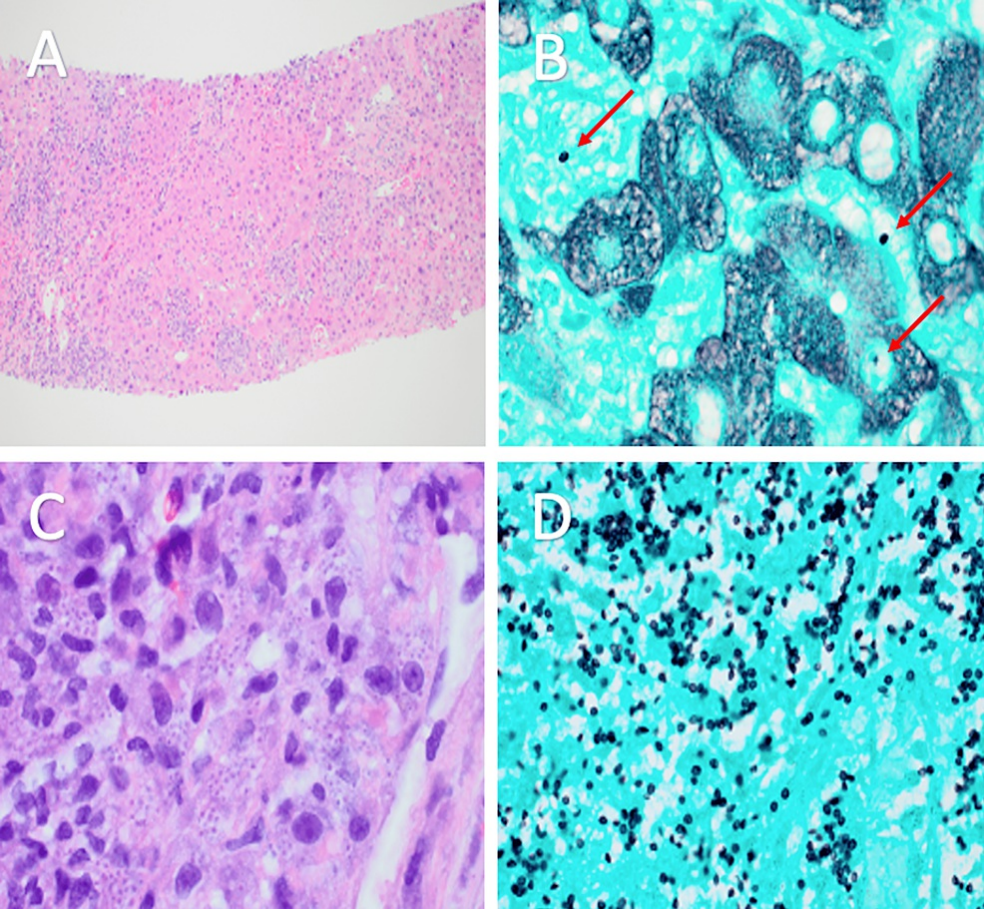
Biopsy specimens of the liver and left adrenal gland (A) Liver biopsy specimen with brisk lymphohistiocytosis and small granulomatous inflammation in the lobules (H&E, 100x). (B) Small yeast forms (red arrows) are present in the granulomas and sinusoidal infiltrate of the liver (GMS, 1000x). (C) Adrenal gland biopsy specimen with numerous intracytoplasmic organisms 3-6 microns with pale halo (H&E, 1000x). (D) Adrenal gland biopsy specimen redemonstrating multiple intracytoplasmic organisms. (GMS, 1000x). H&E: Hematoxylin and eosin, GMS: Grocott methenamine silver

A biopsy of the left adrenal gland under CT guidance demonstrated intracytoplasmic *Histoplasma* species with background necrosis and acute inflammation (Figure 3). The patient’s total bilirubin continued to rise. Ultrasound-guided liver biopsy demonstrated bridging fibrosis in the graft liver likely related to previous recurrent HCV, granulomatous hepatitis due to Histoplasma species, and no evidence of acute cell-mediated rejection (Figure 3). The patient continued to have intermittent fevers and initially received amphotericin for acute disseminated histoplasmosis, but this medication caused an acute kidney injury (AKI). The patient was transitioned to itraconazole instead.

Endocrinology was consulted due to concern for adrenal insufficiency. Prednisone was held in preparation for an Adrenocorticotrophic hormone (ACTH) stimulation test, of which the results supported secondary adrenal insufficiency related to prolonged use of corticosteroids. Although histoplasmosis may have been contributing to the results, the patient should have had little to no adrenal response if the adrenal insufficiency was truly due to histoplasmosis. Prednisone was thereafter restarted. 

**Figure 4:**
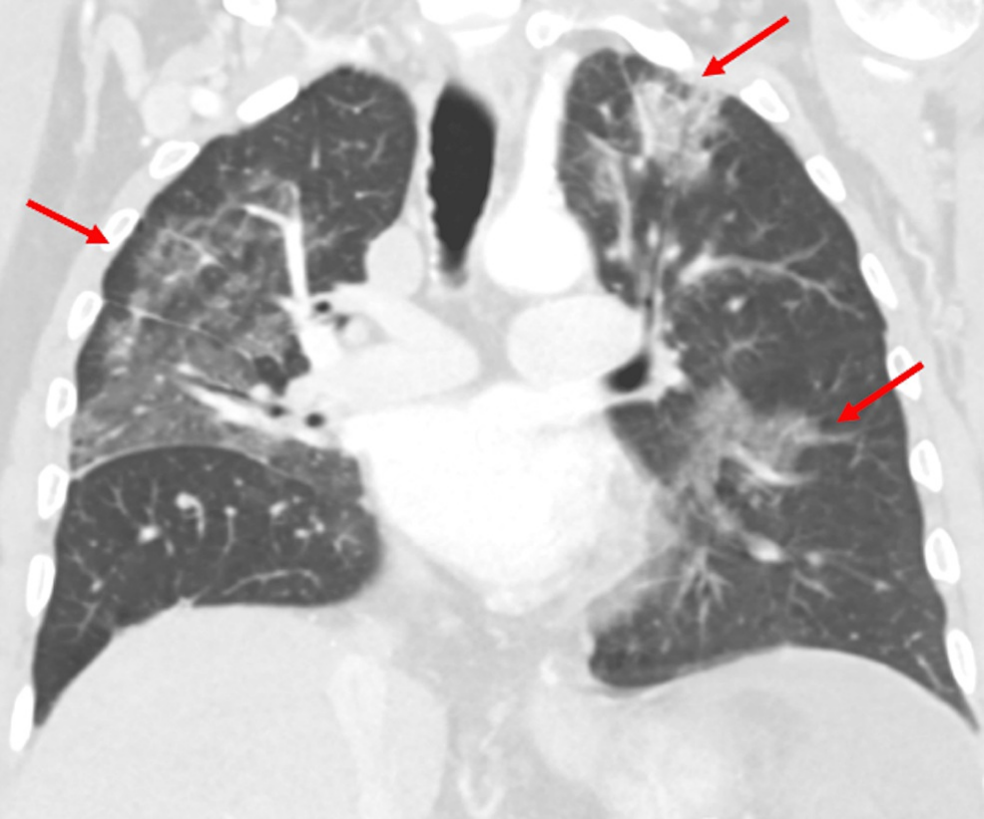
Coronal CT of the chest showing bilateral ground glass opacities Chest CT shows the progression of bilateral multifocal ground glass consolidation (red arrows) in both lungs representing the progression of multifocal pneumonia.

The patient developed hematemesis and melena associated with hypotension, acute hypoxic respiratory failure, and multi-organ failure, requiring a transfer to the ICU. A follow-up CT of the chest demonstrated the progression of multifocal pneumonia in both lungs (Figure 4). An esophagogastroduodenoscopy demonstrated esophageal varices with no active bleeding and multiple aphthous ulcers in the esophagus. As the patient became septic, urine and sputum cultures were found to be positive for *Klebsiella pneumoniae*, and the Infectious Disease consult team recommended meropenem. 

The patient developed poor mentation, and a lumbar puncture was performed. Cerebrospinal fluid studies were negative for organisms. The patient was found to be positive for *Clostridium difficile* antigen and Toxin A + B on a stool test, and treatment with vancomycin contributed to an improved mental status. After a Goals of Care conversation with the family, the patient was transitioned to comfort care measures. Unfortunately, the patient passed away the next morning.

## Discussion

Rapid antigen testing is the gold standard for diagnosis of histoplasmosis in the United States and should have been considered early on in this patient’s hospital course. For countries in which rapid antigen testing is not available, diagnosis can still be achieved by culturing a clinical specimen (e.g., blood, tissue, sputum, pulmonary fluid) to detect intracellular *H. capsulatum*. However, the sensitivity of culture is low and the growth of organisms may take 4-6 weeks. Histological evaluation of organ biopsies may be utilized to rapidly investigate the presence or absence of yeast forms in organs of suspected involvement. The histopathology of histoplasmosis involves granulomatous inflammation with caseating necrosis in tissues such as lymph nodes, liver, bone marrow, skin, or mucous membranes in patients with disseminated infection [1]. In the case of our patient, the initial cultures of blood, urine, and sputum samples and *Blastomyces dermatitidis* quantitative antigen test with blood and urine samples were all negative. Despite negative serology, the diagnosis of disseminated histoplasmosis was ultimately made after histological analysis of the left adrenal gland and liver biopsies showed intracytoplasmic *Histoplasma* species. Physicians must consider various combinations of aforementioned laboratory techniques when suspecting histoplasmosis, as the diagnosis may be elusive. 

Our patient had multiple risk factors for acute disseminated histoplasmosis, including long-term residency in the Midwestern United States and immunocompromised status due to organ transplantation (including liver and kidney) and splenectomy. A literature search on PubMed revealed 45 case reports describing bilateral adrenal histoplasmosis, but most of these articles discuss the diagnosis and disease course in immunocompetent hosts. Additionally, these cases reported the presence of primary rather than secondary adrenal insufficiency if hypoadrenalism was involved. Previous literature has also found that most cases of histoplasmosis regardless of adrenal involvement occur within the first two years after solid organ transplantation [3]. Asplenic patients are also prone to histoplasmosis. Hence, healthcare providers should encourage splenectomy patients to wear masks for preventing exposure to *H. capsulatum* if they live in endemic areas and anticipate contact with soil [7]. Overall, our case report is unique as it describes a delayed presentation of bilateral adrenal histoplasmosis and secondary adrenal insufficiency at 21 years after the initial liver transplant in an immunocompromised host. 

Adrenal histoplasmosis with or without hypoadrenalism rarely occurs in immunocompetent hosts, as it typically presents as a self-limited infection unless the individuals have been exposed to a large dose of inoculum. In individuals with appropriate innate immune responses, the infection is usually cleared from the body within two weeks. Though if symptoms do manifest, patients with adrenal histoplasmosis may present with non-specific symptoms of fatigue, weight loss, nausea, and orthostasis for months. To differentiate fungal infection from the malignancy of the adrenal gland, the adrenal biopsy gland may be particularly useful, especially in immunocompetent individuals [8]. Typical imaging findings of adrenal histoplasmosis on CT or MRI may involve bilateral adrenal enlargement, peripheral enhancement, and central hypodensity due to necrosis and/or hemorrhage [5]. 

Among developed countries, opportunistic and fungal infections represent 10% of primary adrenal insufficiency cases [9]. In cases of adrenal hypofunction, adrenal histoplasmosis should be included in the differential diagnosis. Pertinent labs and vitals of adrenal insufficiency include hypoglycemia and orthostatic hypotension, and physical exam findings may include hyperpigmentation [6]. Disseminated histoplasmosis with adrenal involvement rarely causes hypoadrenalism and adrenal failure, though the incidence rates of these complications are unknown. A Kauffman et al. study found that only 12 out of 58 elderly patients with histoplasmosis had involvement of the adrenal glands. None of these patients developed adrenal failure [5]. Unfortunately, as patients tend to present with non-specific symptoms, adrenal histoplasmosis as a cause of adrenal insufficiency is often a missed diagnosis and a diagnostic challenge in non-endemic areas and immunocompetent individuals. In our presented case, an adrenocorticotropic hormone (ACTH) stimulation test demonstrated that the patient had hypoadrenalism related to long-term use of corticosteroids rather than due to adrenal histoplasmosis. Therefore, this is an atypical presentation of hypoadrenalism in the setting of disseminated histoplasmosis, as the patient presented with secondary rather than primary adrenal insufficiency. At the same time, our case supports the statement that hypoadrenalism and adrenal failure are rare complications of disseminated histoplasmosis with the involvement of the adrenal glands.

Liver findings in the context of disseminated histoplasmosis are seen in 90% of patients. However, hepatic histoplasmosis rarely occurs without primary lung manifestations, which was the case for our patient as well. The most common finding of hepatic histoplasmosis from the liver biopsy is portal lymphohistiocytic inflammation. Granulomatous hepatitis, as in the case of our patient, is found in less than 20% of cases of hepatic histoplasmosis [10]. Interestingly, though our patient’s abdominal CT did not show evidence of disseminated liver infection, the continued rise in total bilirubin prompted a liver biopsy which demonstrated yeast forms. As suggested from the overall course of diagnosis and treatment, in this case, physicians must consider a holistic evaluation of laboratory and imaging studies in conjunction with histology for investigating infectious etiologies. 

Per the Infectious Disease Society of America’s 2007 guidelines for the management of histoplasmosis, therapy can range from conservative management to lifelong suppressive therapy with itraconazole depending on the patient’s immune function and the severity of the infection. In untreated patients with disseminated histoplasmosis, the mortality can be as high as 80%-100%; however, the mortality rate can be reduced to less than 25% with antifungal treatment [5]. More aggressive therapy, including amphotericin B followed by itraconazole, is needed in cases of disseminated histoplasmosis. Antigen levels should be measured at the therapy initiation and at 12 months after the conclusion of therapy to monitor for potential relapse [11]. Treatment failure or relapse was suspected in our patient, for which reasons may include poor absorption of itraconazole, non-adherence to medications, or resistance to antifungal treatments [12]. 

## Conclusions

Histoplasmosis is often a self-limited, asymptomatic infection in immunocompetent individuals. However, this fungal infection can present as disseminated histoplasmosis with multisystem organ involvement and complications in immunosuppressed patients. Within appropriate clinical contexts, physicians should include histoplasmosis as a differential diagnosis in both immunocompetent and immunocompromised patients in nonendemic and endemic areas. A delayed presentation of histoplasmosis should be considered if patients develop symptoms many years after solid organ or stem cell transplant, prolonged use of immunosuppressive drugs, or HIV infection. The initial gold standard test for suspected histoplasmosis is rapid antigen detection of *H. capsulatum*, but diagnosis may still be achieved with culturing or histological examination of biological specimens. When histoplasmosis is suspected or known, imaging tests such as chest X-ray, CT of the lungs, and/or CT of the abdomen may have utility in evaluating the status and progression of the disease. Biopsy of organs and consequent histological analysis may be performed to determine the presence or absence of yeast forms. Adrenal involvement in disseminated histoplasmosis should be considered in the event of adrenal hypofunction and especially when the adrenal glands are enlarged on imaging tests. Treatment of symptomatic histoplasmosis involves initiation of itraconazole with monitoring of antigen levels. More aggressive therapy is required in cases of disseminated histoplasmosis, including amphotericin B followed by itraconazole.
